# AGTR1 rs3772622 gene polymorphism increase the risk of nonalcoholic fatty liver disease patients suffer coronary artery disease in Northern Chinese Han population

**DOI:** 10.1186/s12944-016-0279-3

**Published:** 2016-06-24

**Authors:** Yang Liu, Lin-Lin Lu, De-Xi Yuan, Ning Geng, Shi-Ying Xuan, Yong-Ning Xin

**Affiliations:** Department of Gastroenterology, Qingdao Municipal Hospital, School of Medicine, Qingdao University, Qingdao, China; Department of Gastroenterology, Qingdao Municipal Hospital, 1 Jiaozhou Road, Qingdao, 266021 China; Digestive Disease Key Laboratory of Qingdao, Qingdao, Shandong Province China; Central Laboratories, Qingdao Municipal Hospital, Qingdao, Shandong Province China; Department of Anorecta, Qilu Hospital, of Shandong University, Qingdao, China

**Keywords:** AGTR1, Polymorphism, Genetic, Non-alcoholic Fatty Liver Disease, Coronary Artery Disease

## Abstract

**Background:**

Cardiovascular disease (CAD) responsible and nonalcoholic fatty liver disease (NAFLD) are both metabolic diseases, and they are mostly influenced by genetic factors. The aim of our study is to evaluate the relationship between angiotensin II type-1 receptor (*AGTR1*) gene *rs3772622* polymorphisms and the risk of developing coronary artery disease (CAD) in Chinese patients with NAFLD.

**Methods:**

Genotype for *AGTR1 rs3772622* in 574 NAFLD patients with CAD or 589 NAFLD patients without CAD, 332 CAD patients exclude NAFLD and 338 health control subjects were determined by sequencing and polymerase chain reaction analysis. Relevant statistical methods were employed to analyze the genotypes, alleles and the clinical date. Inter-group differences and associations were assessed statistically using *t*-*tests* and *Chi* square and logistic analyses. The relative risk of *AGTR1 rs3772622* for NAFLD was estimated by logistic regression analysis.

**Results:**

No significant difference in genotype and allele frequency of *AGTR1 rs3772622* was found between the NAFLD without CAD population and the controls (P > 0.05). However, makeable difference was found when compared the CAD in patients with NAFLD and CAD free NAFLD patients (*P* < 0.001 OR = 2.09). Similarly, significant difference was found in *AGTR1 rs3772622* genotype distribution between the groups of CAD patients and control (*P* = 0.046 OR = 1.71).

**Conclusions:**

*AGTR1 rs3772622* gene polymorphism was not associated with the risk of NAFLD, but could increase the risk of NAFLD patients suffering from CAD in the Chinese Han population. Deeply mechanisms underlying the association between *AGTR1 rs3772622* gene polymorphism and the risk of CAD in NAFLD patients need more research.

## Background

Nonalcoholic fatty liver disease (NAFLD) influenced by the incidence of Obesity has become one of the most common chronic liver disease and a primary health issue [1]. NAFLD is a metabolic disease without obvious alcohol intake, because of the abnormal metabolism making fat accumulated in the liver. On behalf of a spectrum of disease, NAFLD range from simple steatosis to steatohepatitis, and finally lead to cirrhosis [2, 3]. NAFLD can increased the risk of all-cause mortality, contributed by liver related deaths as well as non-liver related causes such as malignancy, diabetes and coronary artery disease (CAD) [4]. Epidemiological studies performed in United States and Japan showed that NAFLD can increased the risk of cardiovascular disease (CVD) and is a predictor of CVD independent of the presence of other metabolic syndrome risk factors, such as hypertension, diabetes, dyslipidaemia, insulin resistance (IR) and obesity [5, 6].

Angiotensin II type 1 receptor (AGTR1) gene has been implicated with susceptibility to NAFLD and was reported playing a fibrogenic role in NAFLD. Polymorphism of AGTR1 rs3772622 was reported to be associated with increased fibrosis score [7]. Furthermore, AGTR1 is an important receptor that controlling blood pressure and regulating cardiovascular homeostasis. It was reported that AGTR1 gene (A1166C) polymorphism was associated with hypertension and atherosclerotic stroke [8–11]. It was reasonable to speculate that AGTR1 may be one of the candidate genes in the susceptibility for NAFLD patients with CAD. However, the role of AGTR1 rs3772622 in atherosclerosis has not been elucidated.

This is the first report to investigate the association between *AGTR1 rs3772622* and NAFLD patients with CAD.

## Methods

### Ethics statement

Our study was confirmed by the Ethical Committee of Qingdao Municipal Hospital (Qingdao, China), the reference number: 2015–01. And we prepared a written informed consent form for the participants to confirm that they were willing to participate. This study was carried out on the basis of the principles of the Declaration of Helsinki [12].

### Study subjects

The controls were recruited from the Departments of Gastroenterology and Cardiology of Qingdao Municipal Hospital. All groups of participant were of Northern Han Chinese origin. NAFLD patients were diagnosed by B-type ultrasonography, The diagnosis of NAFLD was according to the standard clinical evaluation [13]. CAD was diagnosed by a percutaneous coronary angiogram, and the final diagnosis was determined by two skilled interventional cardiologists, diagnostic criteria for CAD is that not less than 50 % stenosis in at least one of the coronary arteries. The individuals in control group were confirmed as being normal by echocardiography and laboratory examinations at the identical hospital. Subjects should be excluded other liver diseases, such as nonalcoholic fatty hepatitis (NASH), the drug-induced liver disease, autoimmune liver disease, viral hepatitis, and the Weekly drinking > 140 g, other diseases such as cardiac disorders, concurrent major renal, infectious disease, diabetes mellitus, and malignant disease, and a history of medication. Study questionnaire was used to get the basic clinic pathological information (name, age, etc.).

To test our hypothesis, we finally selected a total of 1163 unrelated adult Chinese NAFLD patients of both genders [574 patients with CAD (292 females, 282 males, mean age 43.81 ± 7.58) and 589 patients without CAD (301 females, 288 males, mean age 45.06 ± 8.77)]. 332 unrelated adult Chinese CAD patients without NAFLD of both genders (168 females, 164 males, mean age 46.45 ± 9.68), and 338 health control of both genders (173 females, 165 males, mean age 43.85 ± 8.79). All subjects were of Northern Han Chinese origin, as described previously.

### Clinical and laboratory assessments

Each of the subjects was fasted for 12 h before examination. For biochemical analyses, blood samples of all participants were determined for Triglyceride (TG), serum levels of total cholesterol (TC), high-density lipoprotein (HDL), low-density lipoprotein (LDL), alanine aminotransferase (ALT), and aspartate aminotransferase (AST), and gamma-glutamyl transpeptidase (GGT). The biochemical tests were analyzed by applying an automatic biochemistry analyzer (Hitachi P7600). Body weight and height were measured for calculate body mass index (BMI). According to a standard protocol of the 3-day baseline observation on each morning, three BP measurements can be obtained. The men who have a systolic BP ≧ 140 mmHg and/or diastolic BP ≧ 90 mmHg or use of antihypertensive medication can be diagnosed as hypertension.

### Genotyping

Simples of peripheral blood were collected and genomic DNA was extracted by using the Genomic DNA Purification Kit (CWBIO, China), then stored at −20 °C until use. The *AGTR1* SNP was genotyped using PCR analysis with the following primers for *AGTR1 rs3772622* polymorphism: 5′ - CAGGAGCTCTGAGAGGAATGTTCAG - 3′ and 5′-TGGCAGATCAGCTGGGTTCATT-3′. The amplified reaction system and the PCR amplification profile progressed as previously described [14].

### Statistical analysis

SPSS statistical software, version 20.0 (SPSS, Chicago, IL, USA) can be used to analyze data. Inspection level a = 0.05. Firstly, the quantitative data in the indexes were tested by *t* test, and the results were represented by the form of mean ± standard deviation (*S.D*.). Through counting DNA sequencing data, the genotype and allele frequencies can be estimated. The distinctions between studied groups were analyzed by Pearson’s χ^2^ test. Then, logistic regression analysis was used to measure the strength of the association between *AGTR1 rs3772622* polymorphism and *CAD*, and it evaluated by the OR (95 % *CI*). At last, by comparing the *OR* values of the two groups with/without *NAFLD*, we can find the association between *AGTR1* gene polymorphisms and coronary artery disease in patients with nonalcoholic fatty liver disease.

## Results

### Sample basic information

Table 1 presented the basic characteristics of this study population. There was no statistical significance in gender and age in each group (all *p* > 0.05). While, compared to the health control group, groups of NAFLD patients, with or without CAD (CAD+ NAFLD/CAD- NAFLD), and CAD patients without NAFLD (NAFLD- CAD) had increased serum ALT, AST, TG, TC, and LDL levels, BMI index, while the HDL levels decreased. (*p*1,2,3 < 0.001).

**Table 1:** Basic characteristics of this study population

Characteristics	Groups				*P* value			
	*CAD*+ *NAFLD* (*n* = 574)	*CAD*- *NAFLD* (*n* = 589)	*NAFLD*-*CAD* (*n* = 332)	Controls (*n* = 338)	P1	P2	P3	P4
Age (years)	43.81 ± 7.58	45.06 ± 8.77	46.45 ± 9.68	43.85 ± 8.79	0.996	0.921	0.884	0.912
Sex Female/male	292/282	301/288	168/164	173/165	0.927	0.981	0.880	0.934
Smoker, no. (%)	244 (42.5)	158 (26.8)	132 (39.6)	105 (31.3)	<0.001	<0.001	0.019	<0.001
Hypertension, no. (%)	279 (48.6)	225 (38.2)	147 (44.3)	71 (21.2)	<0.001	<0.001	<0.001	<0.001
ALT (U/L)	41.05 ± 8.50	40.69 ± 9.42	21.91 ± 5.90	19.15 ± 6.04	<0.001	<0.001	<0.001	0.487
AST (U/L)	42.67 ± 7.58	42.32 ± 8.12	22.11 ± 5.71	21.63 ± 5.82	<0.001	<0.001	0.035	0.451
GGT (U/L)	40.18 ± 8.42	40.08 ± 8.42	22.37 ± 5.35	19.43 ± 5.60	<0.001	<0.001	<0.001	0.839
TG (mmol/L)	2.49 ± 0.94	2.02 ± 0.82	1.41 ± 0.75	1.28 ± 0.70	<0.001	<0.001	0.027	<0.001
TC (mmol/L)	5.50 ± 1.01	4.72 ± 1.32	4.52 ± 1.22	4.04 ± 1.24	<0.001	<0.001	<0.001	<0.001
HDL (mmol/L)	1.10 ± 0.25	1.22 ± 0.31	1.34 ± 0.46	2.19 ± 0.64	<0.001	<0.001	<0.001	<0.001
LDL (mmol/L)	3.22 ± 0.62	2.96 ± 0.72	2.40 ± 0.57	2.54 ± 0.62	<0.001	<0.001	<0.001	<0.001
BMI (kg/m^2^)	26.88 ± 3.77	26.29 ± 4.03	26.18 ± 3.42	23.22 ± 2.69	<0.001	<0.001	<0.001	0.988

*BMI* body mass index, *ALT* alanine aminotransferase, *AST* aspartate aminotransferase, *TG* triglyceride, *TC* total cholesterol, *HDL* high-density lipoprotein, *LDL* low-density lipoprotein. p_1_: *CAD*+ *NAFLD* vs. Control; p_2_: *CAD*-*NAFLD* vs. Control; p_3_: *NAFLD*-*CAD* vs. Control; p_4_: *CAD*+ *NAFLD* vs. *CAD*-*NAFLD*

Importantly, CAD+ NAFLD group patients had higher TG, TC, and LDL levels and lower HDL level than CAD- NAFLD group patients (*p*4 < 0.001).

### *AGTR1 rs3772622* genotype and allele distribution

The genotype and allele distribution were showed in Table 2, it indicated that there was no significant difference between CAD- NAFLD patients and control group (*p* > 0.05). As shown in Table 3, there was significant difference in genotypic and allelic distributions between the NAFLD patients with or without CAD (OR: 2.09, 95 % CI: 1.49-2.95, *P* < 0.001; OR: 1.83, 95 % CI: 1.39-2.41, *P* < 0.001 respectively). Furthermore, a considerable significant difference of genotype distribution were observed between CAD patients without NAFLD and health control people (OR: 1.71, 95 % CI: 1.002-2.860, *P* = 0.046), While there’s no significant difference of alleles distribution were observed from the above groups (OR: 1.04, 95 % CI: 0.77-1.41, *P* = 0.80).

**Table 2:** Distribution of*AGTR1* rs772622 polymorphisms in *CAD*-*NAFLD* and control groups

Groups	genotype				alleles
	T/T	C/T	C/C	T	C
control	115 (34)	155 (45.9)	68 (20.1)	385 (56.9)	291 (43.1)
*CAD*-*NAFLD*	161 (27.3)	301 (51.1)	127 (21.6)	623 (52.4)	555 (46.4)
χ^2^	4.64			2.86	
*p* value	0.098			0.091

**Table 3:** Distribution of *AGTR1* rs772622 polymorphisms in these study groups

Genotype	*CAD*+ *NAFLD* N (%)	*CAD*-*NAFLD* N (%)	*NAFLD*-*CAD* N (%)	Controls N (%)	OR (95%CI)	P1	OR (95%CI)	P2
TT	168 (29.3)	259 (44.0)	147 (44.3)	153 (45.3)				
CT	292 (50.9)	246 (41.8)	129 (38.9)	155 (45.8)				
CC	114 (19.8)	84 (14.2)	56 (16.8)	30 (8.9)	2.09 (1.49-2.95)	<0.001	1.71 (1.02-2.86)	0.046
AlleleT	628 (54.7)	764 (64.9)	400 (60.2)	461 (68.2)				
AlleleC	520 (45.3)	628 (54.7)	264 (39.8)	215 (31.8)	1.83 (1.39-2.41)	<0.001	1.04 (0.77-1.41)	0.80

P1: *CAD*-*NAFLD* vs *CAD* + *NAFLD*; P2: *CAD* + *NAFLD*- vs control

## Discussion and conclusions

In China, NAFLD has become the most common form of liver disease, and it recognized as the second largest chronic liver disease [15–18]. As a hepatic manifestation of the metabolic syndrome, we can suppose that NAFLD may promote the development of atherosclerosis [5, 6]. Some reports demonstrated that because of the promoting effect on CVD, NAFLD patients were at high risk of death [8, 10]. Nowadays many epidemiological and clinical studies focus on the association between NAFLD and CAD [19–24]. Several studies have reported that NAFLD is a strong risk factor for CAD [25, 26]. A study demonstrates that NAFLD is an independent risk factor for angiographically in Koreans population, and also demonstrate that in Asians NAFLD can independently influence the progression to CAD [26].

*AGTR1* is the receptor of Angiotensin II (AngII) [27], AngII as a potent vasopressor hormone and a main regulator of aldosterone secretion plays an important role in blood pressure control and also related to the pathogenesis of coronary diseases [11, 28–31]. The main effect of *AGTR1* is that can promote vascular contraction, regulating blood pressure and promoting aldosterone release [27]. Al-N et al. found that AGT rs2067853ˎrs699ˎ rs3789679ˎ rs2148582ˎ rs5051 polymorphisms could increase the risk for myocardial infarction [32].

Recently, people found that angiotensin II seemed to have a key character in the development of NASH [7]. They found that AngII acts on *AGTR1* to activate hepatic stellate cells (HSCs), which could express the transforming growth factor-β1 (TGF-β1). TGF-β1 worked as a counter response to liver injury [33], While Z et al. found that *AGTR1* gene *rs3772622* was not associated with NAFLD and NASH [17]. This was in contrast with the Japanese study. Our studies found that *AGTR1* gene *rs3772622* was not associated with NAFLD in Chinese Han. The cause of the difference between different populations may be related to the factors such as race, environment and NAFLD diagnostic criteria. Angiotensin II type1 (AT1) receptors were divided into AT1a and AT1b two subtypes, and the distribution of subtype in liver tissue was racial difference. The distribution of AT1 subtype in Chinese population has not clear yet.

The current study is the first report to report that *AGTR1* polymorphism increased the risk of NAFLD patients suffering from CAD. In this study we demonstrated that when compared with TC and TT genotype, the CC homozygote and carriers of C allele was significantly associated with NAFLD in patients with CAD, by comparing the risk between NAFLD patients with or without CAD, we found that there was a statistically significant difference in patients with CC genotype. then comparing the risk between without NAFLD people with or without CAD, we found that there still had statistically significant difference in people with CC genotype. However, by comparing the two OR values, *AGTR1 rs3772622* polymorphisms can increase the risk of CAD in patients with NAFLD (OR1 > OR2).

NAFLD and CAD are both metabolic diseases, and study found that NAFLD is an independent risk factor for CAD. There must be a mechanism for the interaction between them.

However, our current study still has several limitations yet. We have the limitations on the studies of NAFLD spectrum, as we all know that the gold standard to diagnose NAFLD is liver biopsy, it can valid ascertainment and scoring of the different NAFLD stages. In clinical diagnosis, only limited application can we use liver biopsy technology. So it could not be widely used in the collection of specimens. Compared with the diagnosis of NASH, diagnosis of NAFLD can be based on radiological, clinical, and laboratory findings. Through these pathways above, it was more easily to collect the specimens of NAFLD patients. Ethics committee stipulated that it is not allow for biopsy in control group, thus no biopsies were applyed in controls. Strict criteria are required to reduce the likelihood of misclassifying in control group.

This final conclusion is that AGTR1 rs3772622 gene polymorphism was not associated with the risk of NAFLD, but could increase the risk of NAFLD patients suffering from CAD in the Chinese Han origin.
